# Existential Anxiety of Nurses in the COVID‐19‐Virus Units and Its Relation With Resilience and Posttraumatic Growth

**DOI:** 10.1002/hsr2.70547

**Published:** 2025-03-06

**Authors:** Asma Ghonchehpour, Farshid Rafiee Sarbijan Nasab, Fatemeh Maghsoudi, Roghayeh Mehdipour‐Rabori

**Affiliations:** ^1^ Student Research Committee Kerman University of Medical Sciences Kerman Iran; ^2^ Nursing Research Center Kerman University of Medical Sciences Kerman Iran

**Keywords:** COVID‐19, existential anxiety, nurse, posttraumatic growth, resilience

## Abstract

**Aim:**

The study aimed to investigate the relationship between existential anxiety, posttraumatic growth, and resilience in nurses working in COVID‐19units.

**Design:**

The study was a descriptive‐analytical study.

**Methods:**

The researchers conducted the study on 224 nurses working in the COVID‐19units of four hospitals affiliated with Kerman University of Medical Sciences in Southeast Iran from 2021 to 2020 with census method. the data were collected using a demographic questionnaire, Masoudi Sani et al.'s existential anxiety questionnaire, the Conner‐Davidson resilience scale, posttraumatic growth inventory.

**Results:**

The mean age of nurses were 30.88% ± 6.53% and 76.3% of them were female. The results showed there were a negative and significant correlation between posttraumatic growth and resilience (*p* < 0.001, *r* = −0.38) but no statistically significant relationship between existential anxiety, resilience, and posttraumatic growth (*p* > 0.05). Regression analysis indicated a negative and significant relationship between posttraumatic growth and resilience, but no statistically significant relationship between existential anxiety, resilience (*p* = 0.28), and Posttraumatic growth (*p* = 0.20). There was no significant difference between the mean existential anxiety score, age, sex, and education level, but the mean existential anxiety score in the emergency personnel was significantly higher than of other departments.

**Conclusion:**

The results demonstrated that the mean scores of existential anxiety, resilience and posttraumatic growth were moderate. The researchers suggest educational and interventional measures to improve resilience, posttraumatic growth and reduce existential anxiety among nurses.

## 1 Introduction

COVID‐19 has spread over the last few decades as one of the most serious threats to public health around the world [1] and put unprecedented work pressure on healthcare workers [2] Nurses are the front line of caring for patients with COVID‐19 [3]. Stressors for healthcare workers include uncertainty about disease transmission and severe infection with the COVID‐19 virus, which can lead to anxiety and Posttraumatic stress disorder in nurses [4].

Remembering COVID‐19‐related death reveals existential anxiety. It is latent but becomes active when confronted with death [5]. Existential anxiety emerges because of inevitable existential issues when humans face the phenomenon of death despite their desires for purpose and immortality [6]. There are three types of anxiety: death and fate, meaninglessness and emptiness, and guilt and condemnation (the quality of life that does not conform to universal standards) [7].

Anxiety disorders are one of the consequences of traumatic experiences. Anxiety disorders are marked by symptoms such as excessive worry, social and performance anxieties, panic attacks that are unexpected or triggered, fear of future events, and avoidance behaviors [8]. However, some stressful situations can pave the way for individuals' progress; traumatized people return to their previous states and reach a higher level of mental performance [9]. Nurses working on the frontlines of COVID‐19 demonstrate dedication and empathy, yet they are endangering their lives while fulfilling their responsibilities [10]. Healthcare workers can develop posttraumatic growth by saving their lives and improving the outcomes of patients [11]. Posttraumatic growth (PTG) is described as “positive psychological transformations that occur following the experience of trauma or extremely difficult circumstances.” This concept should be viewed not as a replacement, but as a complementary process alongside negative psychological outcomes [12]. PTG was accure during the process of coping with anxiety [13]. Nurses suffer from distress, obsession, and mind rumination because of witnessing COVID‐19‐related deaths and concerns about their families' infection. According to a recent study, posttraumatic growth affects perceived distress [14]. Yuanyuan et al. demonstrated that the posttraumatic growth of nurses who faced the COVID‐19 epidemic was at a high level [10]. But the results of Zadafshar et al.'s research in Iran, showed that the posttraumatic growth level of nurses in the COVID‐19 virus patient care unit was lower than the average level [15].

Resilience is effective in controlling and reducing individuals' anxiety in critical conditions, including the COVID‐19 outbreak [16]. Resilience involves preserving adequate functioning, preventing underlying stressors, and effectively adjusting to challenging life situations. Hence, it is beneficial not only to feel secure when encountering obstacles [17]. Nurses caring for patients must have a high level of resilience and manage their work stress and moral challenges during the COVID‐19 outbreak [18]. Manzanares et al. demonstrated that nurse professionals had a high degree of resilience despite the overwhelming nature of the COVID‐19 pandemic [19]. Jamebozorgi et al. in Iran, showed that nurses had a moderate level of resilience During the COVID‐19 Pandemic [20].

Barzillay et al. (2020) found that higher resilience scores and lower levels of anxiety and depression were associated with increased resilience during the COVID‐19 pandemic among healthcare and nonhealthcare workers [21]. Tomaszek et al. examined the impact of posttraumatic stress on posttraumatic growth through existential anxiety and life satisfaction levels during COVID‐19 [5]. Ghaedi‐Heidar et al. discovered that greater resilience and lower anxiety were linked to increased posttraumatic growth in individuals in Iran [3].

Nurses suffer from posttraumatic stress disorder, existential anxiety, and distress because of witnessing mortality and the consequences of the epidemic; posttraumatic growth improves these symptoms [22]. Low levels of resilience associates with feelings of mental pressure, anxiety, and depression [23]. Improving resilience in nurses reduces their mental and emotional problems and increases their mental health. Nurses have an important role in maintaining and promoting health, so their anxiety disorders have negative effects on their psychological health and they must improve their resilience and posttraumatic growth. Therefore, the present study was designed and carried out.

**Objectives:**

1. To assess the levels of existential anxiety experienced by nurses working in COVID‐19 units.
2. To measure the resilience levels of nurses working in COVID‐19 units.
3. To measure the posttraumatic growth levels of nurses working in COVID‐19 units.
4. To explore the relationship between existential anxiety and resilience among nurses in COVID‐19 units.
5. To explore the relationship between existential anxiety and and posttraumatic growth among nurses in COVID‐19 units.
6. To explore the relationship between resilience and and posttraumatic growth among nurses in COVID‐19 units.
7. To explore the relationship between existential anxiety, resilience, and posttraumatic growth among nurses in covid‐19 virus units: a regression analysis approach.

**Research questions:**

1. What are the levels of existential anxiety experienced by nurses working in COVID‐19 units?
2. What are the resilience levels of nurses working in COVID‐19 units?
3. What are the posttraumatic growth levels of nurses working in COVID‐19 units?
4. Is there a relationship between existential anxiety and resilience among nurses in COVID‐19 units?
5. Is there a relationship between existential anxiety and posttraumatic growth among nurses in COVID‐19 units?
6. Is there a relationship between resilience and posttraumatic growth among nurses in COVID‐19 units?
7. How do existential anxiety, resilience, and posttraumatic growth relate to each other among nurses in COVID‐19 virus units? Can this relationship be analyzed using regression analysis?

## 2 Methods

### 2.1 Participants

The researchers used the census method in this study. During the research, the following nurses were working in different departments of Afzalipour hospital: 40 nurses in the emergency department, 18 nurses in the COVID‐19 ICU, 35 nurses in the infectious wards, 20 in the suspected ward, 20 in the General ICU, 20 in the COVID‐19 Wards, 15 in the pulmonary ward, and 16 in the gastrointestinal department. Sixty, thirty, and fourteen nurses were working in emergency departments of Shafa, Bahonar, and Shahid Beheshti hospitals, respectively (*N* = 288). The inclusion criteria were nurses' willingness to participate in the research, with a work experience in COVID‐19 wards for at least 3 months, without severe depression and anxiety disorders, and no addiction to narcotics and psychotropic drugs. The exclusion criteria were employee turnover and incomplete questionnaires. We excluded the participants who did not complete more than a third of the questionnaires.

The researchers invited 288 nurses to participate in the study, but 235 completed the questionnaire (response rate = 81%). 11 incomplete questionnaires were excluded, so 224 nurses participated in this research. The mean age of nurses was 30.88 ± 6.53. 76.3% of them were female and 57.6 percent were married. 51% of them worked in Emergency ward. 46% of them were infected once with COVID‐19. Table 1 illustrates nurses' demographic information.

**Table 1:** The distribution of the participants in terms of the demographic characteristics.

Variable	Mean (SD)
**Age**	**30.88 ± 6.53**
	***N* (%)**
**Gender**	
Female	171 (76.3)
Male	53 (23.7)
**Marital status**	
Single	88 (39.3)
Married	129 (57.6)
Divorced	7 (3.1)
**Education**	
Diploma	11 (4.9)
Bachelor	201 (89.7)
Master	12 (5.4)
**Name of wards**	
Emergency ward	115 (51.3)
Infectious	32 (14.3)
Suspected ward	19 (8.5)
General ICU	11 (4.9)
COVID‐19 ICU	18 (8.1)
Pulmonary	12 (5.4)
Gastrointestinal	10 (4.5)
Psychiatric	6 (2.7)
**Infection with COVID‐19**	
0	73 (32.6)
1	103 (46.0)
2	43 (19.2)
3	5 (2.2)
**Having information about COVID‐19**	
No	10 (4.5)
Yes	212 (95.5)
**Source of information**	92 (41.1)
Iran health ministry	
WHO	32 (14.4)
Social media	117 (52.5)
Partners	99 (44.4)
In‐service training	26 (11.7)
Other ways	21 (9.4)
**Family members with COVID‐19 infection**	
No	61 (27.4)
Yes	162 (72.6)
**Caring for family members**	
No	29 (13.1)
Yes	193 (86.9)
**Death of a family member with COVID‐19**	
No	185 (82.6)
Yes	39 (17.4)

### 2.2 Measures

#### 2.2.1 Demographic Information Questionnaire

This questionnaire included information about participants' age, sex, marital status, work experience in nursing, education level, name of the department, frequency of COVID‐19 infection, sources used to obtain information about the disease, enough information about the diagnosis, prognosis, and treatment of COVID‐19, death of close relatives with COVID‐19, caring for family members with COVID‐19.

#### 2.2.2 Existential Anxiety Questionnaire

The researchers used an existential anxiety questionnaire developed by Masoudi Sani et al. [27]. The 29‐item questionnaire contains four subscales: death anxiety [4, 8, 11, 15, 19, 23, 24], responsibility anxiety [1, 5, 9, 13, 16, 20, 25, 26], loneliness anxiety [2, 6, 10, 14, 17, 21], and meaning anxiety [3, 7, 10, 12, 18, 22, 27]. The items are graded on a 4‐point scale ranging from one (not at all) to four (very much). The scores vary between 29 and 116, with a low score indicating ignoring existential anxiety and a high score indicating experiencing high existential anxiety. To interpret the scores, in this study, the scores were divided into three groups: low [11, 28, 29, 30, 31, 32, 33, 34, 35, 36, 37, 38, 39, 40, 41, 42, 43, 44, 45, 46, 47, 48, 49, 50, 51, 52, 53, 54, 55, 56], moderate (58.1–87) and high (87.1–116). Masoudi Sani et al. used the intraclass correlation coefficient (ICC) method to report the content validity (0.95) as well as concurrent and convergent validity of the questionnaire (0.82 and 0.55, respectively). They reported the questionnaire reliability to be 0.82 and 0.86 using Cronbach's alpha and retest methods, respectively [27]. In the present study, Cronbach's alpha coefficient was obtained at 0.76.

#### 2.2.3 Conner‐Davidson Resilience Scale (CD‐RIS)

CD‐RIS developed by Conner‐Davidson and Mohammadi et al. [26] translated into Persian. This 25‐item scale was used to measure nurses' resilience based on a Likert scale ranging from zero (not true at all) to four (true nearly all of the time). These ratings result in a number between 0 and 100, and higher scores indicate higher resilience. Conner‐Davidson reported the resilience scale to be 0.89 using Cronbach's alpha coefficient [25]. In Iran, Mohammadi et al. (2006) standardized the resilience scale; they confirmed its validity and calculated its reliability (0.89) using Cronbach's alpha coefficient [26]. In the present study, Cronbach's alpha coefficient was obtained at 0.91.

#### 2.2.4 Posttraumatic Growth Inventory (PTGI)

Tedeschi and Calhoun (1996) developed this 21‐item inventory on a six‐point Likert scale ranging from zero (I did not experience this change as a result of my crisis) to five (I experienced this change to a very great degree as a result of my crisis). The scores range from 0 to 105, with the higher score indicating greater posttraumatic growth [24]. Tedeschi and Calhoun reported the internal consistency of this inventory to be 0.90 [28]. In Iran, Seyed Mahmodi et al. (2013) translated into Persian. Seyed Mahmodi et al. (2013) used Cronbach's alpha coefficient to report reliability for the whole inventory (0.92) and they confirmed its validity and reliability [29]. The reliability of subscales was between 0.55 and 0.75 In the present study, Cronbach's alpha coefficient was obtained at 0.92.

### 2.3 Study Design and Setting

The descriptive‐correlational cross‐sectional study was conducted on nurses working in COVID‐19 units of teaching hospitals affiliated with the Kerman University of Medical Sciences in 2021. Kerman, a city in southeast Iran. Kerman is the largest province in Iran. It has four big teaching hospitals affiliated with the Kerman University of Medical Sciences. Kerman also is a ceter of referral of neighboring provinces. The researchers chosed COVID‐19 units in Afzalipur, Shahid Bahonar, Shafa, and Shahid Beheshti hospitals. Afzalipur is the COVID‐19 center of Kerman, with the highest number of departments intended for people with COVID‐19 infection.

### 2.4 Procedure Carried Out

The researchers started sampling after obtaining permission from hospitals affiliated with Kerman University of Medical Sciences. They selected nurses working in COVID‐19 wards by census method. They had to do online sampling due to the spread of COVID‐19 and nurses' insufficient time. First, they prepared an electronic questionnaire, then explained the research objectives to the supervisors of each department and invited them to participate in the study. The researchers could access and contact eligible nurses and explain to them the research objectives, the research method, and how to fill in the electronic questionnaire. The researchers referred the participants to the questionnaire link to read and sign the consent form; Nurses received the link to the questionnaire on WhatsApp and had enough time to complete it.

### 2.5 Data Analysis

the researchers used the SPSS25 trial version to analyze the data. Descriptive statistics (frequency, percentage, mean and standard deviation) were used to describe the background characteristics of nurses, existential anxiety, resilience, and posttraumatic growth. Kolmogorov–Smirnov test was used to check the normality of the data. Pearson correlation coefficient was used to determine the relationship between existential anxiety, resilience, and posttraumatic growth. linear regression analysis was used to determine the relationship between existential anxiety, resilience, and posttraumatic growth. As data were normally distributed with equal variances, independent *t* and ANOVA tests were employed to compare scores of existential anxiety, resilience, posttraumatic growth, and demographic variables. A significance level of 0.05 was considered.

## 3 Results

### 3.1 Descriptive

The mean age of nurses were 30.88% ± 6.53% and 76.3% of them were female. We assessed the normality of the variables, including existential anxieties and its dimensions, resilience, and posttraumatic growth of nurses working in COVID‐19 wards, and confirmed that they were normally distributed. The normality of the data was verified using the Kolmogorov–Smirnov test.

Table 2 indicates the mean scores of existential anxieties and its dimensions, resilience, and posttraumatic growth of nurses working in COVID‐19 wards. The mean score of existential anxiety was 80.92 ± 9.18 and it was at moderate level (cut point = 58.1–87), the mean resilience score was a little higher than the median score (=50), and the mean posttraumatic growth score was equal to the median score (=87).

**Table 2:** The descriptive statistics for existential anxiety and its dimensions, resilience, and posttraumatic growth.

Variable	Mean ± SD
Existential anxiety	
Death anxiety	20.40 ± 3.14
Responsibility anxiety	22.96 ± 3.81
Loneliness anxiety	15.79 ± 2.50
Meaning anxiety	18.64 ± 2.89
Total	80.92 ± 9.18
Total resilience	63.51 ± 15.00
Total posttraumatic growth	87.69 ± 16.55

### 3.2 Correlational

Table 3 illustrates the relationship between existential anxiety, resilience, and posttraumatic growth. There were a negative and significant correlation between posttraumatic growth and resilience (*p* < 0.001, *r* = −0.38) but no statistically significant relationship between existential anxiety, resilience, and posttraumatic growth (*p* > 0.05) (Table 3).

**Table 3:** Correlation between existential anxiety, resilience, and posttraumatic growth.

Variable	Pearson coloration		
	Existential anxiety	Resilience	Posttraumatic growth
**Existential anxiety**	1	0.08	−0.09
***p* value**		0.28	0.20
**Resilience**	−0.09	1	−0.38
***p* value**	0.20		< 0.001
**Posttraumatic growth**	0.08	−0.38	1
***p* value**	0.30	< 0.001	

### 3.3 Prediction

Table 4 illustrates the relationship between existential anxiety, resilience, and posttraumatic growth. Regression analysis showed that there was a negative and significant correlation between posttraumatic growth and resilience but no statistically significant relationship between existential anxiety, resilience (*p* = 0.28), and posttraumatic growth (*p* = 0.20) (Table 4).

**Table 4:** The relationship between resilience and posttraumatic growth.

Model	Unstandardized coefficients		Standardized coefficients	*t*	Sig.
	B	Std. Error	Beta		
1 (Constant)	113.88	4.62		24.65	0.00
Resilience	−0.42	0.07	−0.38	−5.87	0.00

*Note:* Dependent Variable: PTG.

### 3.4 Comparison

The researchers noticed a significant difference between the department and the mean scores of existential anxiety and resilience, so the mean score of existential anxiety in emergency personnel was higher than that in personnel of COVID‐19 units, but resilience was higher in those working in other departments than those in the emergency department. The result was shown a significant difference between the death of relatives and existential anxiety, so that the total mean score of anxiety was 84.17 ± 9.09 for those whose one of their relatives died of COVID‐19, while it was 80.24 ± 9.09 for those whose one of their relatives did not die of COVID‐19 (*p* < 0.02). There was no significant difference between other demographic variables and the mean scores of existential anxiety, resilience, and posttraumatic growth (*p* > 0.05).

## 4 Discussion

The study aimed to investigate the relationship between existential anxiety, resilience, and posttraumatic growth in nurses working in COVID‐19 wards of the hospitals affiliated with Kerman University of Medical Sciences.

The results indicated that the mean score of existential anxiety in nurses was at moderate level. The results were consistent with the results of Huang et al. (2020) who stated that nurses experienced anxiety, fear, and sadness during the COVID‐19 outbreak. The disease was a source of anxiety for different social groups, particularly those who were exposed to the disease [30]. Silwal et al. (2020) demonstrated high levels of anxiety in nurses [31]. Korkmaz et al. (2020) concluded that healthcare workers developed psychological disorders, such as anxiety during the COVID‐19 pandemic [32]. Sarboozi Hosein Abadi et al. (2020) revealed moderate levels of depression, anxiety, and stress in nurses working in COVID‐19 wards in Nohom Day hospital in Torbat Heydarieh [33]. But, Ghaedi‐Heidari et al. (2022) concluded that the medical students had a lower level of anxiety in COVID‐19 pandemic [3]. In general, their results suggested moderate prevalence or high levels of psychological disorders, particularly anxiety in nurses during the COVID‐19 outbreak. Anxiety is the most basic characteristic of crises that disturbs social and individual life structures [34]. Nurses in COVID‐19 wards are at risk of developing psychological disorders due to the nature of their work, heavy protective clothing, N‐95 masks, and the risk of becoming infected and contaminating others [35, 36].

The results showed that the mean resilience score was a little higher than the median score and it was almost at moderate level. Some other studies showed a moderate level of resilience among nurses during the COVID‐19 pandemic [37, 38, 39]. Roberts et al. (2021) in the United Kingdom, Luceño‐Moreno et al. (2020) in Spain, and Yusefi et al. (2021) found moderate levels of resilience among nurses during the COVID‐19 pandemic [40, 41, 42]. Ghaedi‐Heidari et al. (2022) demonstrated that the medical students had a high level of resilience in COVID‐19 pandemic [3]. However, Afshari et al. (2021) reported a low resilience score in nurses during the outbreak of COVID‐19 [43]. The COVID‐19 spread had a negative impact on the resilience of people; the sample size, the research population, and the hospital management atmosphere were among the causes of this difference. The researchers recognized that optimal resilience assisted healthcare workers in exhibiting more adaptive behaviors in stressful situations, such as the spread of COVID‐19 and the high volume of admitted patients [44], so nurses and doctors could accept the existing conditions and perform at their best [45]. Resilience refers to positive patterns of adaptation to adversity and problems that arise over time; nurses with favorable resilience will demonstrate greater flexibility and mental health over time [46]. The studies believed that resilient healthcare workers cope with psychological pressures and problems, respond to stressors, deal with daily problems, and show better adaptability in stressful situations, such as the COVID‐19 pandemic [47].

The results showed that nurses experienced a moderate level of posttraumatic growth during the COVID‐19 pandemic. Cui et al. (2020) and Peng et al. (2021) reported a moderate level of posttraumatic growth in nurses [48, 49]. Afzali et al. (2020) indicated a moderate level of posttraumatic growth in nurses during the COVID‐19 pandemic [50]. Ghaedi‐Heidari et al. (2022) demonstrated that the medical students had a high level of PTG in COVID‐19 pandemic [3]. Their results are in line with our results. Individuals' occupations require them to endure their jobs; [51] nurses always work in difficult conditions and have high burnout [52]. Nurses from various departments were among the first to fight against the COVID‐19 virus. Nurses and doctors were under a lot of pressure because they were unable to treat COVID‐19. They witnessed the deaths of many patients and the distress of their families, which led to posttraumatic stress and psychological problems in healthcare workers [11, 53, 54, 55]. Nurses and other healthcare workers used various methods to deal with these problems and improve the quality of their care during the COVID‐19 pandemic; they improved their posttraumatic growth through their communication with others and resilience in the face of illness and death.

The results were shown a negative and significant relationship between posttraumatic growth and resilience‐ with higher resilience resulting in lower posttraumatic growth‐ but no statistically significant relationship between existential anxiety, resilience, and posttraumatic growth. Nasirzadeh et al. reported that although the mean scores of depression, anxiety, and stress decreased with the increase in resilience during the COVID‐19 pandemic, they noticed no significant relationship between them [56]. Most of the studies did not support our study. Perhaps, the sample size, the research population, and the hospital management atmosphere were among the causes of this difference. Peng et al. indicated a negative correlation between posttraumatic growth and trait anxiety but a positive correlation between posttraumatic growth and resilience in medical students. Resilience could mediate the relationship between trait anxiety and posttraumatic growth [57]. Atay et al. recognized that there is a positively significant relationship between posttraumatic growth and psychological resilience in nurses working at the pandemic clinics [58]. Barzillay et al. recognized that an increase in the resilience score resulted a decrease in anxiety and depression among healthcare and non‐healthcare workers, and a higher resilience score had an association with less concern about COVID‐19 [21]. Ghaedi‐Heidari et al. found a positive and significant relationship between resilience and posttraumatic growth in medical student [3].

Resilience has a negative effect on posttraumatic growth during the COVID‐19 pandemic, because resilient individuals are flexible, adapt to the environmental changes that occurred by COVID‐19, and quickly recover after the crisis. Therefore, a significant relationship between posttraumatic growth and resilience is expectable. Perhaps, the reasons for a negative relationship between resilience and PTG were related to the samples from different nurses who worked in different wards such as emergency and ICU. Because these patients have different care needs and are in different situations. In addition, the nurses in the study are still involved in caring for patients with covid‐19, and they are still at risk of infection. Perhaps, if the study will be conducted another time after the end of the COVID‐19 epidemic, different results will be found. As the studies have shown, Growth after trauma depends on different factors, and the present study showed the average score of PTG. The researchers think if the scores of PTG were higher, they would find other results, perhaps, a positive relationship between resilience and PTG. So the researchers suggest a similar study accomplished after ending the COVID‐19 pandemic.

## 5 Limitation

One of the limitations of the present study was the collection of information through social media; therefore, people who did not have access to these resources were unable to respond. Another limitation of the study was the study of a small sample of the country and the nurses of Kerman hospitals, so we should generalize the data with caution.

## 6 Conclusion

The results found a negative and significant relationship between posttraumatic growth and resilience‐an increase in resilience resulted in a decrease in posttraumatic growth‐ but no statistically significant relationship between existential anxiety, resilience, and posttraumatic growth. Nurses had moderate existential anxiety, resilience and posttraumatic growth. The researchers suggest training courses and workshops with a focus on controlling and reducing existential anxiety and promoting resilience during the COVID‐19 pandemic to reduce the negative effects of existential anxiety in healthcare workers.

Since the earliest days of the spread of COVID‐19, nurses have dealt with this virus; they were away from their families, lacked proper protective equipment, and witnessed the deaths of a large number of patients, resulting in psychological problems that hindered their productivity. In addition to physical and social care, it is necessary to focus on the prolonged psychological dimensions of COVID‐19 in nurses to promote posttraumatic growth and resilience and reduce existential anxiety among healthcare workers. Limited studies addressed the relationship between resilience, existential anxiety, and posttraumatic growth in nurses during the outbreak of COVID‐19, so we were unable to confirm or reject our results. It is necessary to conduct more studies because demographic and cultural factors and governance systems of hospitals can affect the results of studies. It is suggested to increase the generalizability of these variables in future research in a wider society and consider more variables. It is also suggested to conduct more studies to investigate the relationship between resilience, posttraumatic growth and existential anxiety with other psychological variables such as anxiety, stress, and mindfulness in nurses.

## Author Contributions

**Asma Ghonchehpour:** writing – original draft, writing – review and editing, methodology, data curation, resources. **Farshid Rafiee Sarbijan Nasab:** conceptualization, investigation, writing – original draft, writing – review and editing, methodology, data curation. **Fatemeh Maghsoudi:** data curation, writing – original draft, writing – review and editing. **Roghayeh Mehdipour‐Rabori:** conceptualization, investigation, writing – original draft, writing – review and editing, visualization, validation, methodology, software, formal analysis, project administration, resources, supervision.

## Ethics Statement

All methods were carried out in accordance with relevant guidelines and regulations of the Kerman University of Medical Sciences. Kerman University of Medical Sciences Ethics committee approved this project (IR.KMU.REC.1400.022). After approval, permission was issued to the management of the hospitals. We provided some oral information, including the goals and objectives of the study, the confidentiality and anonymity of the data, and that the participants were free to withdraw from the study at any time. Then written informed consent was taken individually from all subjects.

## Consent

The authors have nothing to report.

## Conflicts of Interest

The authors declare no conflicts of interest.

## Transparency Statement

The lead author Roghayeh Mehdipour‐Rabori affirms that this manuscript is an honest, accurate, and transparent account of the study being reported; that no important aspects of the study have been omitted; and that any discrepancies from the study as planned (and, if relevant, registered) have been explained.

## Data Availability

The datasets used and/or analyzed during the current study are available from the corresponding author on reasonable request.

## References

[1] A. Al‐Rabiaah , M.‐H. Temsah , A. A. Al‐Eyadhy , et al., “Middle East Respiratory Syndrome‐Corona Virus (MERS‐COV) Associated Stress Among Medical Students at a University Teaching Hospital in Saudi Arabia,” Journal of Infection and Public Health 13, no. 5 (2020): 687–691. 32001194 10.1016/j.jiph.2020.01.005 PMC7102651

[2] N. Greenberg and M. G. S. W. Docherty , “Managing Mental Health Challenges Faced by Healthcare Workers During Covid‐19 Pandemic,” BMJ 26, no. 368:m12 (2020): 1–4, 10.1136/bmj.m1211. 32217624

[3] F. Ghaedi Heidari , F. Lohrasbi , N. Toghian Chaharsoughi , S. Samsamshariat , and F. Rafiee Sarbijan Nasab , “The Relationship Between Anxiety, Resilience, and Posttraumatic Growth of the Medical Students in COVID‐19 Pandemic in Iran,” Iranian Journal of Psychiatry and Behavioral Sciences 16, no. 4 (2022): e115333.

[4] M. Leng , L. Wei , X. Shi , et al., “Mental Distress and Influencing Factors in Nurses Caring for Patients With COVID ‐19,” Nursing in Critical Care 26, no. 2 (2021): 94–101. 33448567 10.1111/nicc.12528

[5] K. M.‐C. A. Tomaszek , “Thinking About My Existence During COVID‐19, I Feel Anxiety and Awe‐The Mediating Role of Existential Anxiety and Life Satisfaction on the Relationship Between PTSD Symptoms and Post‐Traumatic Growth 2,” International Journal of Environmental Research and Public Health 27, no. 19 (2020): 7062. 10.3390/ijerph17197062 PMC7579162 32992515

[6] A. Yasaman and H. R. Homayoon , “The Effectiveness of Mindfulness‐Based Cognitive Therapy on Rumination and Existential Anxiety in the Elderly Men,” Journal of Aging Psychology 8, no. 1 (2022): 1–10.

[7] E. P. Galanaki , L. J. Nelson , and F. Antoniou , “Social Withdrawal, Solitude, and Existential Concerns in Emerging Adulthood,” Emerging Adulthood 11, no. 4 (2023): 1006–1021.

[8] K. L. Szuhany and N. M. Simon , “Anxiety Disorders: A Review,” Journal of the American Medical Association 328, no. 24 (2022): 2431–2445. 36573969 10.1001/jama.2022.22744

[9] Z. Asgari and A. Naghavi , “Explaining Post‐Traumatic Growth: Thematic Synthesis of Qualitative Research,” Iranian Journal of Psychiatry and Clinical Psychology 25, no. 2 (2019): 222–234.

[10] Y. Mo , P. Tao , G. Liu , et al., “Post‐Traumatic Growth of Nurses Who Faced the COVID‐19 Epidemic and Its Correlation With Professional Self‐Identity and Social Support,” Frontiers in Psychiatry 12 (2022): 562938. 35095580 10.3389/fpsyt.2021.562938 PMC8794949

[11] R. Chen , C. Sun , J. J. Chen , et al., “A Large‐Scale Survey on Trauma, Burnout, and Posttraumatic Growth Among Nurses During the COVID‐19 Pandemic,” International Journal of Mental Health Nursing 30, no. 1 (2021): 102–116. 33107677 10.1111/inm.12796 PMC7894338

[12] L. Dell'osso , P. Lorenzi , B. Nardi , C. Carmassi , and B. Carpita , “Post Traumatic Growth (PTG) in the Frame of Traumatic Experiences,” Clinical Neuropsychiatry 19, no. 6 (2022): 390–393. 36627947 10.36131/cnfioritieditore20220606 PMC9807114

[13] F. Vescovelli , S. Minotti , and C. Ruini , “Exploring Post‐Traumatic Growth in Parkinson's Disease: A Mixed Method Study,” Journal of Clinical Psychology in Medical Settings 28, no. 2 (2020): 267–278. 10.1007/s10880-020-09713-9 32144615

[14] P. Wang , K. Wang , Z. Ping , and P. C. C. Wang , 2021, Occupational & Environmental Medicine 78(129–35). 10.1136/oemed-2020-106540 33060188

[15] S. Zadafshar , M. Kheradmand , H. Kazemian , and N. Akrami , “Predicting Covid‐19 Traumatic Stress and Post‐Traumatic Growth in Nurses of Coronavirus Patient Care Unit Based on Perceived Social Support: The Mediating Role of Self‐Compassion and Cognitive Emotion Regulation,” Journal of Applied Psychology Research 13, no. 3 (2022): 327–341.

[16] M. C. Yap , F. Wu , X. Huang , et al., “Association Between Individual Resilience and Depression or Anxiety Among General Adult Population During COVID‐19: A Systematic Review,” Journal of Public Health 4 (2023): e639–e655, 10.1093/pubmed/fdad144. 37580860

[17] A. Ghonchehpour , M. A. Forouzi , M. Dehghan , A. Ahmadi , G. Okou , and B. Tirgari , “The Effect of Mindfulness‐Based Stress Reduction on Rejection Sensitivity and Resilience in Patients With Thalassemia: A Randomized Controlled Trial,” BMC Psychiatry 23, no. 1 (2023): 281. 37085765 10.1186/s12888-023-04802-z PMC10119526

[18] S. Jo , S. Kurt , J. A. Bennett , et al., “Nurses' Resilience in the Face of Coronavirus (COVID‐19): An International View,” Nursing & Health Sciences 23, no. 3 (2021): 646–657. 34169629 10.1111/nhs.12863 PMC8447204

[19] I. Manzanares , S. Sevilla Guerra , M. Lombraña Mencía , N. Acar‐Denizli , J. Miranda Salmerón , and G. Martinez Estalella , “Impact of the COVID‐19 Pandemic on Stress, Resilience and Depression in Health Professionals: A Cross‐Sectional Study,” International Nursing Review 68, no. 4 (2021): 461–470. 34097305 10.1111/inr.12693 PMC8242605

[20] M. H. Jamebozorgi , A. Karamoozian , T. I. Bardsiri , and H. Sheikhbardsiri , “Nurses Burnout, Resilience, and Its Association With Socio‐Demographic Factors During COVID‐19 Pandemic,” Frontiers in Psychiatry 12 (2022): 803506. 35095618 10.3389/fpsyt.2021.803506 PMC8795765

[21] R. Barzilay , T. M. Moore , D. M. Greenberg , et al., “Resilience, COVID‐19‐Related Stress, Anxiety and Depression During the Pandemic in a Large Population Enriched for Healthcare Providers. 2020;10(1):8–10,” Translational Psychiatry 10, no. 1 (2020): 8–10. 32820171 10.1038/s41398-020-00982-4 PMC7439246

[22] T. Tian‐Ci Quek , W. Wai‐San Tam , B. X. Tran , et al., “The Global Prevalence of Anxiety Among Medical Students: A Meta‐Analysis,” International Journal of Environmental Research and Public Health 16, no. 15 (2019): 2735. 31370266 10.3390/ijerph16152735 PMC6696211

[23] S. Samani and B. S. N. Jokar , “Effects of Resilience on Mental Health and Life Satisfaction,” Iranian Journal of Psychiatry and Clinical Psychology 13, no. 3 (2007): 290–295.

[24] M. R. M. S. f. Heidarzadeh , “Dimensions of Posttraumatic Growth in Patients With Cancer,” Middle East Journal of Cancer 5, no. 1 (2014): 23–29.

[25] S. Karimi and M. Esmaeili , “The Relationship Between Emotion Regulation and Resilience Strategies With Marital Adjustment in Female Teachers,” Journal of Psychological Science 19, no. 87 (2020): 291–298.

[26] M. Mohammadi , A. Jazayeri , A. Rafie , and B. P. A. Joukar , “Resilience Factors in Individuals at Risk For Substance Abuse,” Journal of Psychology 1, no. 2–3 (2006): 203–224.

[27] R. Masoudi Sani , B. Bahmani , A. Asgari , and M. P. N. Khanjani , “Construction, Validity and Reliability of Existential Anxiety Questionnaire (Qfea),” Clinical Psychology Perspective 13, no. 25 (2016): 151–162.

[28] R. G. Tedeschi and L. G. Calhoun , “The Posttraumatic Growth Inventory: Measuring the Positive Legacy of Trauma,” Journal of Traumatic Stress 9, no. 3 (1996): 455–471. 8827649 10.1007/BF02103658

[29] S. Seyed Mahmoodi , C. H. Rahimi , and N. Mohamadi , “Psychometric Properties of Posttraumatic Growth Inventory in an Iranian Sample,” Psychological Models and Methods 3, no. 12 (2013): 93–108.

[30] L. R. L. H. Huang , “Emotional Responses and Coping Strategies of Nurses and Nursing College Students During COVID‐19 Outbreak,” MedRxiv 15, no. 8 (2020): e0237303. 10.1371/journal.pone.0237303 PMC7413410 32764825

[31] M. Silwal , D. Koirala , S. Koirala , and A. Lamichhane , “Depression, Anxiety and Stress Among Nurses During Corona Lockdown in a Selected Teaching Hospital, Kaski, Nepal,” Journal of Health and Allied Sciences 10 (2020): 82–87.

[32] S. Korkmaz , A. Kazgan , S. Çekiç , A. S. Tartar , H. N. Balcı , and M. Atmaca , “The Anxiety Levels, Quality of Sleep and Life and Problem‐Solving Skills in Healthcare Workers Employed in COVID‐19 Services,” Journal of Clinical Neuroscience 80 (2020): 131–136. 33099335 10.1016/j.jocn.2020.07.073 PMC7425768

[33] T. Sarboozi Hosein Abadi and M. M. K. N. Askari , “Depression, Stress and Anxiety of Nurses in COVID‐19 Pandemic in Nohe‐Dey Hospital in Torbat‐e‐Heydariyeh City, Iran,” J Mil Med 22, no. 6 (2020): 526–553.

[34] M. Saffarinia , “The Prediction of Mental Health Based on the Anxiety and the Social Cohesion That Caused by Coronavirus,” Social Psychology Research 9, no. 36 (2020): 129–141.

[35] C.‐Y. Liu , Y.‐z Yang , X.‐M. Zhang , X. Xu , and Q. L. Z. W.‐W. Dou , “The Prevalence and Influencing Factors for Anxiety in Medical Workers Fighting COVID‐19 in China: A Cross‐Sectional Survey,” Epidemiology and Infection 20, no. 148 (2020): e98. 10.1017/S0950268820001107 PMC7251286 32430088

[36] M. Heidarijamebozorgi , H. Jafari , R. Sadeghi , et al., “The Prevalence of Depression, Anxiety, and Stress Among Nurses During the Coronavirus Disease 2019: A Comparison Between Nurses in the Frontline and the Second Line of Care Delivery,” Nursing and Midwifery Studies 10, no. 3 (2021): 188.

[37] S. Jose and M. C. M. Dhandapani , “Burnout and Resilience Among Frontline Nurses During COVID‐19 Pandemic: A Cross‐Sectional Study in the Emergency Department of a Tertiary Care Center, North India,” Indian Journal of Critical Care Medicine 24, no. 11 (2020): 1081–1088. 33384515 10.5005/jp-journals-10071-23667 PMC7751034

[38] X. Ou , Y. Chen , Z. Liang , S. Wen , S. Li , and Y. Chen , “Resilience of Nurses in Isolation Wards During the COVID‐19 Pandemic: A Cross‐Sectional Study,” Psychology, Health & Medicine 26, no. 1 (2021): 98–106. 33305600 10.1080/13548506.2020.1861312

[39] D. Afshari , M. Nourollahi‐Darabad , and N. Chinisaz , “Demographic Predictors of Resilience Among Nurses During the COVID‐19 Pandemic,” Work 68, no. 2 (2021): 297–303. 33492260 10.3233/WOR-203376

[40] N. J. Roberts , K. McAloney‐Kocaman , K. Lippiett , E. Ray , L. Welch , and C. Kelly , “Levels of Resilience, Anxiety and Depression in Nurses Working in Respiratory Clinical Areas During the Covid Pandemic,” Respiratory Medicine 176 (2021): 106219. 33248362 10.1016/j.rmed.2020.106219 PMC7648185

[41] L. Luceño‐Moreno , B. Talavera‐Velasco , Y. García‐Albuerne , and J. Martín‐García , “Symptoms of Posttraumatic Stress, Anxiety, Depression, Levels of Resilience and Burnout in Spanish Health Personnel During the COVID‐19 Pandemic,” International Journal of Environmental Research and Public Health 17, no. 15 (2020): 5514. 32751624 10.3390/ijerph17155514 PMC7432016

[42] A. R. Yusefi , S. Daneshi , E. R. Davarani , P. Nikmanesh , G. Mehralian , and P. Bastani , “Resilience Level and Its Relationship With Hypochondriasis in Nurses Working in COVID‐19 Reference Hospitals,” BMC Nursing 20 (2021): 219. 34727947 10.1186/s12912-021-00730-z PMC8561347

[43] D. Afshari , M. Nourollahi‐darabad , and N. Chinisaz , “Demographic Predictors of Resilience Among Nurses During the COVID‐19 Pandemic,” Work (Reading, Mass.) 68 (2021): 297–303. 33492260 10.3233/WOR-203376

[44] Y. M. Aljehani , S. A. Othman , N. K. Telmesani , et al., “Stress and Psychological Resilience Among General Surgery Residents During COVID‐19 Pandemic,” Saudi Medical Journal 41, no. 12 (2020): 1344–1349. 33294893 10.15537/smj.2020.12.25577 PMC7841594

[45] S. Yörük and D. Güler , “The Relationship Between Psychological Resilience, Burnout, Stress, and Sociodemographic Factors With Depression in Nurses and Midwives During the COVID‐19 Pandemic: A Cross‐Sectional Study in Turkey,” Perspectives in Psychiatric Care 57, no. 1 (2021): 390–398. 33103773 10.1111/ppc.12659

[46] M. Yıldırım , G. Arslan , and A. Özaslan , “Perceived Risk and Mental Health Problems Among Healthcare Professionals During COVID‐19 Pandemic: Exploring the Mediating Effects of Resilience and Coronavirus Fear,” International Journal of Mental Health and Addiction 20, no. 2 (2022): 1035–1045. 33223977 10.1007/s11469-020-00424-8 PMC7668285

[47] L. Lorente , M. Vera , and T. Peiró , “Nurses Stressors and Psychological Distress During the COVID‐19 Pandemic: The Mediating Role of Coping and Resilience,” Journal of Advanced Nursing 77, no. 3 (2021): 1335–1344. 33210768 10.1111/jan.14695 PMC7753515

[48] X. Peng , H. Zhao , Y. Yang , Z. Rao , D. Hu , and Q. He , “Post‐Traumatic Growth Level and Its Influencing Factors Among Frontline Nurses During the COVID‐19 Pandemic,” Frontiers in Psychiatry 12 (2021): 632360. 34177641 10.3389/fpsyt.2021.632360 PMC8219941

[49] P. Cui , P. Wang , K. Wang , Z. Ping , P. Wang , and C. Chen , “Post‐Traumatic Growth and Influencing Factors Among Frontline Nurses Fighting Against COVID‐19,” Occupational and Environmental Medicine 78, no. 2 (2021): 129–135. 33060188 10.1136/oemed-2020-106540

[50] M. Afzali , S. hajizadeh koli , P. Aber , and N. Ghasemi , “Prediction of Post‐Traumatic Growth Based on Psychological Well‐Being and Mindfulness During Coronavirus Conditions Among Female Nurses,” Journal of Applied Family Therapy 2, no. 9 (2022): 162–175.

[51] W. Cadge , M. Lewis , J. Bandini , et al., “Intensive Care Unit Nurses Living Through COVID‐19: A Qualitative Study,” Journal of Nursing Management 29, no. 7 (2021): 1965–1973. 33930237 10.1111/jonm.13353 PMC8236976

[52] M. Leng , L. Wei , X. Shi , et al., “Mental Distress and Influencing Factors in Nurses Caring for Patients With COVID‐19,” Nursing in Critical Care 26, no. 2 (2021): 94–101. 33448567 10.1111/nicc.12528

[53] M. Murat , S. Köse , and S. Savaşer , “Determination of Stress, Depression and Burnout Levels of Front‐Line Nurses During the COVID‐19 Pandemic,” International Journal of Mental Health Nursing 30, no. 2 (2021): 533–543. 33222350 10.1111/inm.12818 PMC7753629

[54] Y. X. Wang , H. T. Guo , X. W. Du , W. Song , C. Lu , and W. N. Hao , “Factors Associated With Post‐Traumatic Stress Disorder of Nurses Exposed to Corona Virus Disease 2019 in China,” Medicine 99, no. 99 (2020): e20965. 32590808 10.1097/MD.0000000000020965 PMC7328992

[55] G. J. Nowicki , B. Ślusarska , K. Tucholska , K. Naylor , A. Chrzan‐Rodak , and B. Niedorys , “The Severity of Traumatic Stress Associated With COVID‐19 Pandemic, Perception of Support, Sense of Security, and Sense of Meaning in Life Among Nurses: Research Protocol and Preliminary Results From Poland,” International Journal of Environmental Research and Public Health 17, no. 18 (2020): 6491. 32906590 10.3390/ijerph17186491 PMC7559728

[56] M. Nasirzadeh , M. Akhondi , A. Jamalizadeh Nooq , and S. Khorramnia , “A Survey on Stress, Anxiety, Depression and Resilience Due to the Prevalence of COVID‐19 Among Anar City Households in 2020: A Short Report,” Journal of Rafsanjan University of Medical Sciences 19, no. 8 (2020): 889–898.

[57] L. Peng , L. Lan , and C. L. M. Xu , “The Effect of Trait Anxiety on Medical Freshmen's Post‐Traumatic Growth: The Mediating Role of Resilience,” Research Square (2021), 10.21203/rs.3.rs-1046599/v1.

[58] N. Atay , G. Sahin‐Bayindir , S. Buzlu , K. Koç , and Y. Kuyuldar , “The Relationship Between Posttraumatic Growth and Psychological Resilience of Nurses Working at the Pandemic Clinics,” International Journal of Nursing Knowledge 34, no. 3 (2022): 226–235, 10.1111/2047-3095.12397. 36303467

